# Phenotypic and Genomic Characterization of Antimicrobial-Resistant *Neisseria gonorrhoeae* of Public Health Concern

**DOI:** 10.3201/eid3206.260310

**Published:** 2026-06

**Authors:** Johan H. Melendez

**Affiliations:** Division of Infectious Diseases, Department of Medicine, Johns Hopkins University School of Medicine, Baltimore, Maryland, USA

**Keywords:** gonorrhea, Neisseria gonorrhoeae, bacteria, antimicrobial resistance, sexually transmitted infections, ceftriaxone, azithromycin, molecular epidemiology, epidemiologic monitoring, Australia

The increasing rates of antimicrobial resistance (AMR) in *Neisseria gonorrhoeae* globally pose a major threat to the management of gonorrhea (*1*). In the United States and many other developed countries, ceftriaxone, a third-generation cephalosporin, is the recommended therapy for the treatment of uncomplicated gonorrhea (*2*,*3*). In Australia, dual treatment with ceftriaxone and azithromycin is currently recommended (*4*). To mitigate the threat of AMR on public health, surveillance of AMR in *N. gonorrhoeae* is critical for monitoring antimicrobial susceptibility trends, assessing emergence of AMR, and guiding treatment recommendations (*5*).

In this issue, Walker et al. (*4*) describe the results of an integrated phenotypic, epidemiologic, and genomic analysis of *N. gonorrhoeae* isolates collected in New South Wales (NSW), Australia, as part of a comprehensive surveillance program during January 2022–December 2024. The isolates were subjected to antimicrobial susceptibility testing and classified as *N. gonorrhoeae* of public health concern because they displayed either decreased susceptibility (DS) to ceftriaxone (MIC >0.125 mg/L) or high-level resistance (HLR) to azithromycin (MIC >256 mg/L). In NSW, detection of an *N. gonorrhoeae* isolate of public health concern prompts a public health response, including collection of samples from each site of infection for test-of-cure (TOC) and enhanced surveillance protocols for identifying treatment failures, informing of emerging resistance trends, and enhancing AMR surveillance. That type of systematic monitoring for treatment failures helps enhance AMR surveillance practices and is a key component of the World Health Organization global action plan to control the spread and impact of AMR in *N. gonorrhoeae* (*5*).

In the analysis by Walker et al., of the 54 patients with ceftriaxone DS *N. gonorrhoeae*, 46 (85.2%) returned for TOC; of those, 2 (4.3%) returned a positive TOC result by nucleic acid amplification test (NAAT) for *N. gonorrhoeae* >2 weeks after treatment, which is suggestive of treatment failure. The high return rate for follow-up TOC reported in this study is notable, considering that other studies have reported lower return rates of 41%–64% (*6*–*9*). In addition, the prospective evaluation and identification of isolates of public health concern helped to identify treatment failures which might otherwise have remained undiagnosed, suggesting that the targeted public health response approach might be a suitable intervention to identify persons requiring retreatment and prevent onward transmission of ceftriaxone DS *N. gonorrhoeae*.

Despite increasing rates of ceftriaxone-resistant *N. gonorrhoeae* globally (*10*), reporting and confirmation of treatment failure cases remain low; possible causes are inconsistent treatment regimens, limited access to culture diagnostics, and heterogenous definitions of treatment failures (*11*). Furthermore, ceftriaxone resistance, for which MIC breakpoints have not been clearly or uniformly defined globally, might not always result in treatment failure (*11*), which suggests that current ceftriaxone resistance breakpoints might need to be reexamined (*12*). Of note, the Clinical and Laboratory Standards Institute guidelines recommend classifying *N. gonorrhoeae* isolates with ceftriaxone MIC <0.125 mg/L as susceptible and those with MIC >0.5 mg/L as resistant (*13*). In the study by Walker et al., of the patients with ceftriaxone DS (MIC >0.125 mg/L) who underwent TOC, 44/46 (95.7%) returned a negative NAAT result. That finding suggests that dual therapy with ceftriaxone and azithromycin was effective in eradicating almost all infections caused by ceftriaxone DS *N. gonorrhoeae* but does not conclusively demonstrate the clinical significance of the >0.125 mg/L breakpoint for defining ceftriaxone DS or resistance.

The increasing rates of ceftriaxone-resistant *N. gonorrhoeae* globally have been driven by surges of *N. gonorrhoeae* harboring the mosaic *penA-*60 allele in the Asia Pacific region (*10*). In the study by Walker et al., 45% of ceftriaxone DS *N. gonorrhoeae* isolates harbored the *penA-*60 allele; that finding is consistent with global reports that the *penA*-60 allele is the most common genetic variation associated with ceftriaxone DS and resistance (*10*,*14*). Network analysis revealed 5 different ceftriaxone DS clusters, including a large cluster of 13 isolates, which suggested local transmission of ceftriaxone DS *N. gonorrhoeae*. The study also provided evidence of overseas acquisition of ceftriaxone DS *N. gonorrhoeae* and local transmission of those overseas-acquired infections, highlighting the possibility of further emergence of ceftriaxone resistance in Australia through importation of resistant strains.

Walker et al. also reported on cases of *N. gonorrhoeae* with azithromycin HLR, which were primarily from nonheterosexual patients (95%), and patients with a history of sexually transmitted infections (70%); cases were approximately evenly distributed across the anatomic sites (urogenital tract, rectum, and pharynx). Of note, whereas genomic analysis identified 2 separate but connected clusters (17/40 cases) of *N. gonorrhoeae* with HLR, many (19 [47.5%]) cases lacked genetic relatedness, suggesting that different strains of *N. gonorrhoeae* with azithromycin HLR are circulating in NSW, Australia. That finding, together with the post–COVID-19 (2022–2024) increase of gonorrhea cases in NSW caused by azithromycin HLR *N. gonorrhoeae*, is consistent with the evolving global epidemiology of *N. gonorrhoeae* with azithromycin HLR (*15*). Of note, *N. gonorrhoeae* multilocus sequence type (MLST) ST9363, which was the second most common MLST (22.5%, 9/40) of cases with HLR reported by Walker et al, is the most widely disseminated ST globally in *N. gonorrhoeae* with HLR to azithromycin (*15*). Although azithromycin HLR was not associated with treatment failures in that study, the increasing rates of *N. gonorrhoeae* with this phenotype is an example of the rapid and constant evolution of AMR in *N. gonorrhoeae*.

The public health approach described by Walker et al. involving phenotypic, genotypic, and epidemiologic analysis of *N. gonorrhoeae* of public health concern is a practical and effective tool to identify treatment failures, prevent further emergence of ceftriaxone resistance, and ultimately help to preserve the clinical utility of ceftriaxone. Additional public health approaches, including monitoring of treatment failures in pharyngeal gonorrhea, which can be caused by ceftriaxone-susceptible *N. gonorrhoeae*, are necessary to prevent onward transmission of antimicrobial-resistant gonorrhea. Culture-based characterization of AMR in *N. gonorrhoeae* needs to be expanded to establish ceftriaxone resistance breakpoints. Finally, clinical trials are warranted not only to determine the efficacy of ceftriaxone in the era of increasing ceftriaxone resistance but also to better define the relationship between AMR in *N. gonorrhoeae* and treatment failures.
